# The Multifaceted Impact of the SARS-CoV-2 Pandemic on Sexual Health, Function, and Behaviors: Implications for Public Health: A Scoping Review

**DOI:** 10.3390/healthcare13050559

**Published:** 2025-03-05

**Authors:** Gonzalo R. Quintana

**Affiliations:** Departamento de Psicología y Filosofía, Facultad de Ciencias Sociales, Universidad de Tarapacá, Arica 1000007, Arica y Parinacota, Chile; gquintanaz@academicos.uta.cl

**Keywords:** COVID-19, SARS-CoV-2, sexual dysfunction, sexual health, sexual well-being, sexual behavior, public health

## Abstract

Background. The SARS-CoV-2 pandemic had a significant impact on sexual health and human behavior, revealing a widespread decline in sexual function and behaviors. Objective. To summarize these findings and highlight their importance for public health, this article discusses the changes observed in sexual function and behavior during the pandemic, as well as potential explanations for these trends. Methods. This study followed the PRISMA-ScR guidelines, using the keyword search commands: “sexual function” AND (“SARS-CoV-2” OR “COVID-19” OR coronavirus) and “sexual behavior*” AND (“SARS-CoV-2” OR “COVID-19” OR coronavirus) in the Scopus and PubMed databases. The search was conducted on 10 March 2024, including articles published from January 2019 to March 2024. Inclusion criteria required studies focusing on sexual health/function during the SARS-CoV-2 pandemic, excluding non-English articles and non-adult populations. Studies were screened based on relevance, methodological rigor, and sample size, with data extraction focusing on sexual behavior/function metrics. Results were synthesized to identify trends and propose explanatory models. Results. While some individuals experienced reductions in sexual desire and activities, others reported increases, indicating varied individual responses to stressors such as a pandemic. Two hypotheses are presented to explain these changes: terror management theory and the dual control model of sexual response. The critical role of public health in addressing sexual health and well-being needs during a health crisis is discussed, emphasizing the importance of providing clear information, ensuring access to remote sexual health services, and reducing stigma. The need to integrate sexual health into the global response to future health crises is highlighted to ensure a comprehensive approach to human well-being. Conclusions. This review shows the multifaceted impact of the pandemic and social distancing in people’s sexual function and behaviors, underscoring the importance of considering sexual health as an integral part of the emergency health planning and response, to promote the physical and mental well-being of the population during crises such as the SARS-CoV-2 pandemic.

## 1. Introduction

The effects of the SARS-CoV-2 pandemic were pervasive, influencing nearly every aspect of societal and individual life. Global figures for confirmed cases and deaths continue to grow, albeit at a much slower rate following the widespread administration of vaccines [1]. The novel SARS-CoV-2 virus in known to impact the functioning of various organs and systems in a manner similar to previously identified coronaviruses [2,3,4]. The pandemic and social distancing measures had widely documented effects on mental health, such as those related to people’s mood [5,6], mediated by a range of factors. However, one of the most overlooked dimensions of health, both in terms of attention and resources, has been sexual health [7,8,9,10].

Sexual health plays a vital role in people’s overall well-being, making it crucial to understand their sexual function to deliver comprehensive healthcare to people across all ages and backgrounds [11]. In turn, sexual dysfunction is estimated to impact over 40–45% of women of reproductive age globally [12,13], whereas global estimations of erectile dysfunction range between 3–76.5% of the population [14]. Like many other dimensions of people’s lives, the pandemic has also hindered people’s sexual function and almost every studied sexual behavior [15]. Yet, sexual health has not received as much attention as other phenomena amid the pandemic, with insufficient research on the pandemic’s effects [16].

To address that gap, this manuscript offers a review of changes in sexual functioning and behavior observed during the three years of the SARS-CoV-2 pandemic, alongside two tentative explanations for these trends. Finally, the role of public health in sexual health during a pandemic context is briefly discussed.

A scoping review was chosen as the methodological framework to address the broad and multifaceted research question: ’What is the impact of the SARS-CoV-2 pandemic on sexual functioning and behaviors in adults’? Scoping reviews are particularly suited for mapping existing evidence, identifying research gaps, and synthesizing findings from heterogeneous studies, which is critical in an emerging field like the impact of COVID-19 on sexual health. This approach allowed the integration of diverse methodologies, populations, and theoretical perspectives to provide a comprehensive overview of the topic [17]. This review follows the principles outlined in the Joanna Briggs Institute guidelines for scoping reviews [17] to address the following question: What is the impact of the SARS-CoV-2 pandemic on sexual functioning and behaviors in adult men and women?

## 2. Methods

This review was performed in accordance with the PRISMA (Preferred Reporting Items for Scoping Reviews and Meta-Analyses) guidelines (see check list in Supplementary Materials). “Impact” was defined as consistent changes or effects—as well as the absence of them—in quantitative measures. The “context” of this review spans the three years of the SARS-CoV-2 pandemic and the associated social distancing measures.

A bibliographic search was conducted in the Scopus and PubMed databases using the following keyword search commands: “sexual function” AND (“SARS-CoV-2”(MeSH) OR “COVID-19” OR coronavirus), along with “sexual behavior*” AND (“SARS-CoV-2”(MeSH) OR “COVID-19” OR coronavirus). The databases Scopus, PubMed, and Web of Science were selected for their extensive coverage of interdisciplinary research spanning medical, psychological, and social sciences. MeSH terms were employed in PubMed to ensure specificity and coverage, while no preliminary search was deemed necessary. The scope of these databases sufficiently overlaps with others in the field, making additional database inclusion redundant.

The search in Scopus was filtered by year (e.g., titles available between 2019 and 2024), field (e.g., medical and social sciences), and document type (e.g., articles and reviews). Empirical and theoretical studies published before 2019 that were included did not explore SARS-CoV-2 and were used only to contextualize the findings. The search was conducted on 10 March 2024. Potential biases arose in the processes of screening and data extraction in this scoping review since the author conducted those steps. However, strategies to minimize bias were adopted. Predefined inclusion and exclusion criteria were applied to ensure uniformity in selecting the studies. Testing on a sub-sample of studies was performed to refine the process and reduce ambiguity. Standardized templates for data extraction were used to ensure consistency in recording study details and outcomes. Still, the search strategy was not complemented with gray literature or titles in other languages than English.

Data synthesis involved qualitative thematic analysis to identify patterns and trends in sexual health/function changes during the pandemic. The analysis followed three phases: data familiarization, coding, and thematic development. Studies were grouped hierarchically based on phenomena, population characteristics (i.e., gender), methodologies (e.g., study design, when the study was performed during the pandemic), and, finally, key findings (e.g., reduction, increase, both, neither). This approach facilitated a comprehensive narrative synthesis, contextualizing the results within theoretical frameworks like terror management theory and the dual control model.

**Figure 1:**
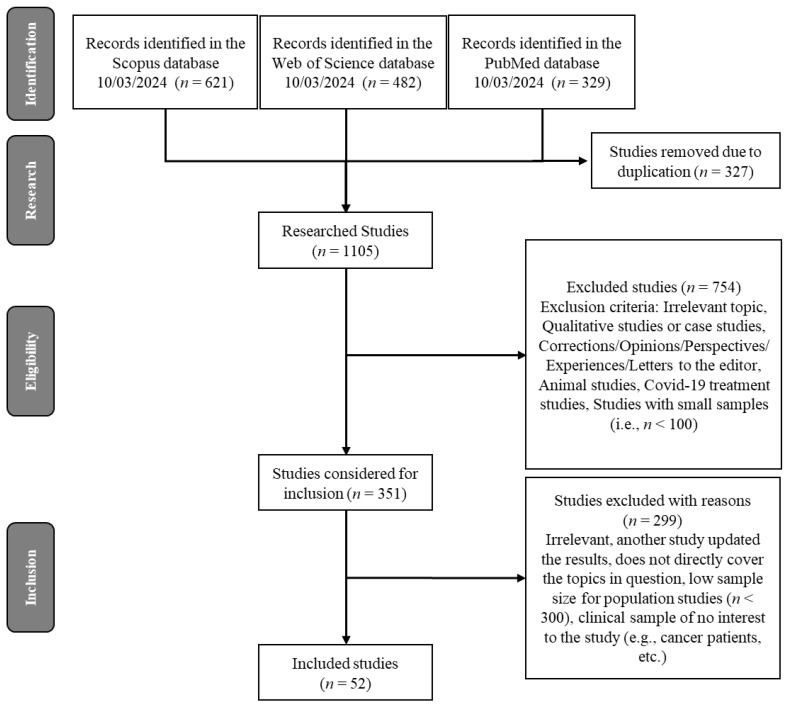
Flowchart of the search, filtering, and selection process of the articles. Adopted from the PRISMA flowchart [17].

Figure 1 presents a flowchart of the search, filtering, and selection process of the articles. Most studies were filtered through the selection and eligibility process based on exclusion criteria, while the chosen studies met the inclusion criteria. Those that best represented the main trends and most consistent findings related to the research question were selected. The chosen studies were more recent, had larger sample sizes (with a few exceptions due to the relevance of the study sample, e.g., pregnant women, people currently or previously infected with SARS-CoV-2, etc.), had less specific populations, and employed more robust statistical analyses. Systematic reviews were also utilized to update the findings and corroborate the main trends. In total, 40 studies were selected to represent general trends addressing the research question, along with other pandemic-related titles discussed throughout this manuscript in its various sections. Only one exception was made for small sample sizes in studies including clinical samples (e.g., with COVID-19) and pre vs. during/post pandemic sexual function or behavioral comparisons.

The definition of sexual health was mostly absent amongst the included studies. Instead, studies relied on the operationalization of sexual function through the use of psychometric instruments.

## 3. Results

A summary of the included studies’ results can be found in Table 1. The thematic analysis yielded four dimensions: 1. Impact on Sexual Function; 2. Changes in Sexual Behavior; 3. Mental Health and Sexuality; 4. Demographic Variations. These were rearranged in the following sections to discuss the thematic characteristics and findings.

### 3.1. Instruments Used and Their Validity

Amongst the selected studies, different instruments were used to measure changes in sexual response, function, and behaviors. For the former, laboratory tests to measure the concentration in fluids were analyzed using either polymerase chain reaction (PCR) or reverse transcription PCR (RT-PCR), which detects and amplifies RNA to identify gene expression [18]. RT-PCR is a sensitive technique, easily a benchmark technology for RNA detection and quantification [19].

Sexual function was measured through psychometric questionnaires. Almost all studies used either the long or short forms of the female sexual function index (FSFI) [20,21] and the international index of erectile function (IIEF) [22,23], as well as the Arizona sexual experiences scale (ASEX) [24], all of which measure sexual desire, arousal, satisfaction, orgasm function, and erectile function/vaginal lubrication, while only the FSFI measures pain, providing consistency in the operationalization of sexual function. Meanwhile, sexual behaviors were solely measured by ad hoc surveys targeting specific behaviors.

Finally, an assortment of different psychometric scales were used to measure their associability with changes in sexual response, function, and behaviors, including measures of symptoms/indicators of depression (e.g., BDI, PHQ-4, PHQ-9), anxiety (e.g., GAD-7), loneliness (i.e., UCLA Loneliness Scale), impulsiveness (i.e., BIS-11), pornography consumption (i.e., PPCS-6), compulsive sexual behaviors (i.e., CSBD-19), etc., or socio-demographic indexes such as the Oxford Stringency Index or the Human Development Index. Most of these instruments are widely used, with ample evidence of the structural and construct validity and reliability of each of them. For instance, recent systematic reviews and meta-analyses for PHQ-4 or PHQ-9 show that they possess strong structural validity evidence, with PHQ-4 showing a stable two-factor structure across demographics and PHQ-9 maintaining diagnostic accuracy, while both exhibit good internal consistency (PHQ-4: α = 0.65–0.88; PHQ-9: reliable across studies) and significant correlations with related measures, supporting construct validity [25,26]. Meanwhile, only a few scales have cross-cultural evidence of their validity and reliability, much less their measurement invariance properties. For instance, PPCS-6 and CSBD-19 have recently been validated across 42 countries, demonstrating excellent validity, reliability, and measurement invariance across genders, sexual orientations, languages, and countries [27,28]. However, most of this information is not made available in the cited studies.

### 3.2. Changes in Sexual Function and Response

Overall, the infection and social distancing fostered by the pandemic predominantly negatively affected various aspects of sexual health [29,30]. The most recent knowledge on the effect of SARS-CoV-2 infection highlights impacts on seminal and testicular function, fertility, and overall sexual health, emphasizing that testicular function typically recovers with disease resolution, in vitro fertilization outcomes are unaffected, and anti-SARS-CoV-2 vaccines help prevent andrological issues [31]. Through assessments of desire, arousal, orgasm, resolution, erectile function, satisfaction, lubrication, and pain, researchers have identified individuals with normal or dysfunctional sexual functioning [32]. For instance, Schiavi et al. [33] reported significant differences in sexual function scores among women before the pandemic and four weeks after the introduction of social distancing restrictions, except in lubrication and pain dimensions. Bhambhvani et al. [34] observed a significant reduction in overall sexual function scores among U.S. women, as well as in the subscales of arousal, lubrication, and satisfaction. Similarly, De Rose et al. [35] conducted a cross-sectional study among Italian hospital workers and non-healthcare workers during the pandemic. Among 544 men and women, they found that male participants had significantly lower scores in satisfaction and desire compared to females, while healthcare workers scored lower only in the sexual satisfaction dimension than non-healthcare workers. A systematic review and meta-analysis of longitudinal studies on female sexual function before and during the SARS-CoV-2 infection corroborated the significantly lower FSFI scores, where only desire and lubrication remained unaffected [36].

**Table 1:** Summary of main results per study.

Author(s) and Year	Sample Size	Study Design and Main Demographics	Measured Outcomes	Measurement Tools	Main Results
Lisco et al. [31]	44	Systematic review of reviews, randomized controlled trials, and meta-analyses	Andrological effects of SARS-CoV-2 infection and COVID-19	-	Eight topics were discussed, including SARS-CoV-2’s effects on seminal changes, male fertility, testosterone levels, and sexual function, as well as the role of vaccines in preventing andrological issues. SARS-CoV-2 impacts testicular function via direct and indirect mechanisms, with severity correlating to dysfunction, though recovery occurs post-disease. IVF outcomes remain unaffected by infection, and erectile and sexual dysfunctions are common due to hormonal imbalances and psychosocial factors related to the pandemic.
Pérez-López et al. [36]	1002	Cross-sectional design; non-pregnant women	Sexual function	FSFI	A meta-analysis of four studies involving 1002 sexually active non-pregnant women found significantly lower FSFI scores during the pandemic compared to pre-pandemic, with reduced scores in arousal −0.80 (−1.13 to −.48), orgasm −0.66 (−1.07 to −0.25), satisfaction −0.59 (−0.97 to −0.22), and pain −0.35 (−0.54 to −0.16), while desire and lubrication remained unaffected. The findings suggest an increased risk of female sexual dysfunction during the pandemic, with results deemed robust and showing a low risk of bias.
Nawaz et al. [37]	300	Longitudinal design; Pakistani women	Sexual function	FSFI	The mean FSFI score for participants before COVID-19 was significantly higher compared to the score 60 days after discharge (28.16 +/− 1.9 vs. 24.43 +/− 2.5; *p*-value: <0.001). Participants who had FSFI score of more than 26 were significantly higher before COVID-19 (72.5% vs. 51.0%; *p*-value: <0.001).
Gacci et al., 2021 [29]	43	Cross-sectional design; adult Italian men	Sexual function, fluid collection	IIEF, RT-PCR	After COVID-19 recovery, 25% of the men had oligo-crypto-azoospermia, with 8 out of 11 being azoospermic and 3 oligospermic. IL-8 was high in 76.7% of the patients, and the severity of COVID-19 was significantly associated with the presence of oligo-crypto-azoospermia (*p* < 0.001).
Pennanen-Iire et al., 2021 [30]	92 studies	Narrative review	Pandemics’ impact on intimacy, sexual health	-	Coronavirus is largely spread through inhalation and infected surfaces, and no sexual transmission has been documented. However, certain sexual behaviors, particularly those involving asymptomatic carriers, raise the risk of spread. Non-monogamy is noted as a common factor in the transmission hubs, hence bringing new challenges to dating and intimacy during the pandemic.
Schiavi et al., 2020 [33]	89 patients	Cross-sectional design; Italian women, living with their partner, without COVID-19 infection	Sexual function change during the social restriction period	FSFI	After the restrictive measures were in place, sexual activity and function significantly decreased, as evidenced by a significant decrease in the frequency of sexual intercourse, sexual function (FSFI), and increased sexual distress (FSDS). General health also deteriorated, reflected in changes in SF-36. Factors such as working outside the home, higher education, and having children were associated with worse sexual function; these factors, when combined, independently predicted lower sexual health.
Bhambhvani et al., 2021 [34]	91	Cross-sectional design; adult women in USA	Differences in pre- and intra-pandemic sexual function	FSFI and PHQ-4	Sexual function in general was significantly lowered due to the pandemic, especially in terms of arousal, lubrication, and satisfaction, but sexual frequency did not change. Sexual dysfunction as reported by RFSD significantly increased during the pandemic period. Women who developed RFSD during the pandemic had higher anxiety and depression compared to others. The development of sexual dysfunction was independent of factors such as age, relationship status, mask-wearing habits, and job changes related to the pandemic.
de Rose et al., 2021 [35]	544	Cross-sectional design; adult Italian men and women	Predictors of depressive symptoms and low sexual desire and satisfaction	FSFI, IIEF, BDI	Significant differences in sexual desire, satisfaction, and depressive symptoms were observed between males and females. Healthcare workers had a higher rate of low sexual desire. Predictors of lower sexual desire included female gender, healthcare worker status, having children, living with a partner, and low sexual satisfaction.
Litam and Lenz [38]	262	Cross-sectional design; young men and women in USA	Sexual behaviors and attitudes, and estimations of mental health	PHQ-4, BSAS, Barratt Impulsiveness Scale	Breaking shelter-in-place orders to pursue sexual activities with partners residing outside the home during the COVID-19 pandemic may be understood as an intentional strategy among men with less favorable birth control attitudes to mitigate the effects of depression.
Hensel et al. [39]	19,654	Cross-sectional design, multi-country men and women	Changes in solo and partnered sexual behaviors with a casual or stable partner	Oxford Stringency Index, Human Development Index, and the Palma Ratio	The most common behavior to increase was hugging, kissing, or cuddling with a partner (21.5%), and the most common behavior to decrease was sex with the main partner (36.7%). Household factors like job/income instability and having children over the age of 12 years were significantly associated with decreased affectionate and partnered sexual behaviors; more frequent substance use was linked to significantly increased solo, partnered, and virtual sexual behaviors.
Ates et al., 2021 [40]	62	Cross-sectional design; Turkey, adult men	Pre- and post-pandemic sexual function and behaviors	IIEF, IELT, PEDT	The average number of weekly sexual intercourses significantly decreased during the pandemic. ED scores were lower, while orgasmic function, sexual satisfaction, general satisfaction, and PEDT scores were higher. Full-time employment and low education levels were identified as risk factors for ED and PE. The negative impact of the pandemic on sexual life was more evident in participants above 65 years. Although several sexual behaviors decreased, such as kissing and face-to-face sex, kissing and face-to-face positions remained the most preferred. There was no significant reduction in risky sexual behaviors among most participants.
Vedovo et al., 2022 [6]	2543	Cross-sectional design; Italian citizens of legal age	Changes in sexual function over and at the end of social restriction	BD, GHS, FSFI, IIEF, UCLA Loneliness Scale	At baseline, 2.6% reported depressive symptoms, 7.4% high levels of loneliness, and 19.4% low general mental health. Sexual dysfunction was reported by 59.1% of men and 68.4% of women. Sexual function did not change significantly over time; however, those sexually active at baseline showed a decline in sexual function scores during the period of social restriction.
Seehuus et al., 2024 [41]	1313	Cross-sectional design; women in USA	Sexual function of those with short, long, and without COVID-19	FSFI, DASS-21	The “never-COVID” group reached higher scores on the FSFI subscales of Desire, Arousal, Lubrication, and Satisfaction compared to the “only-COVID” and “long-COVID” groups. The “only-COVID” group reached significantly higher scores regarding arousal, lubrication, and orgasm but lower scores for pain and overall sexual function when compared with the “long-COVID” group. The FSFI total score was also higher for the “never-COVID” group. All the proposed mediation models showed a less than adequate fit.
de Oliveira et al., 2021 [42]	34 articles	Narrative review, 18 countries	Current findings	Survey	These studies documented a decline in women’s sexual function related to the pandemic, specifically related to sexual desire, frequency of intercourse, and sexual satisfaction. Quite a few women reported an increase in solitary sexual behavior and low relationship satisfaction. In such cases, gender inequalities contributed significantly to lowering sexual function and satisfaction and could widen the pleasure gap between men and women.
Masoudi et al., 2022 [15]	21 studies	Systematic review and meta-analysis, 2454 women and 3765 men	Effects of COVID-19 pandemic on sexual activity and functioning	FSFI, IIEF-5	Sexual functioning and activity were measured by the FSFI for women and by the IIEF-5 for men. The FSFI score among women was significantly lowered during the pandemic, with an SMD of −4.26, indicating a significant decline in sexual function. The IIEF-5 score was also significantly lowered among men, with an SMD of −0.66. Both were statistically significant.
Karagöz et al., 2020 [43]	21 studies	Cross-sectional design; Turkey, adult men and women	COVID-19 pandemic effects on sexual activity and functioning	FSFI, IIEF	The meta-analysis revealed a significant decline in sexual function during the COVID-19 pandemic: in women, the score of FSFI decreased with the SMD of −4.26, and that of IIEF-5 also decreased with SMD of −0.66 in men. Most studies reported that sexual activity was reduced, with increased solo sexual behaviors (especially masturbation) compared with the time before the COVID-19 pandemic.
Pérez et al., 2022 [44]	1986	Cross-sectional design; adult Brazilian Spanish and Portuguese men and women	Sexual function	EEIF-5, FSFI, IES-R	A sample of 1986 participants was studied, out of which 743 reported E/S dysfunction. The incidence of PTSD was higher among E/S dysfunction participants, with males having an IES-R score of 26.54 and females having an IES-R score of 35.92. Participants not living with a partner were 74% more likely to develop E/S dysfunction, while living with a partner during the pandemic had a greater positive effect on sexual function.
Omar et al., 2021 [45]	479	Cross-sectional; adult Egyptian females and males	Effect of COVID-19 pandemic on sexual satisfaction	FSFI, IIEF-5, GAD-7, PHQ-9, Index of sexual satisfaction	Sexual satisfaction during the lockdown was significantly lower for both males (70.5%) and females (56.2%) compared to before the lockdown (91.2% for males, 73.5% for females). Males were more satisfied with their sexual performance than females. Whereas the majority of males (68.2%) did not have any erectile dysfunction, almost all females (97.3%) showed sexual difficulties. In women, the prevalence of sexual stress was 70.8%, and it was 63.1% in males. Occupation, anxiety, erectile dysfunction, and education were significant determinants for male sexual stress. Housewife status, husband’s age, marriage duration, anxiety, and sexual dysfunction were identified to predict sexual stress in female patients.
Bulut et al., 2021 [46]	359	Cross-sectional; healthcare professionals in USA during COVID-19	Severe erectile dysfunction	IIEF-5, IES-R	Health workers, especially those in the Diagnosed Patient Area, had a significantly higher level of stress disorder and erectile dysfunction compared to other groups, with *p* < 0.001. The median IIEF-5 was significantly higher for nurses, those who were married, and those working in the Diagnosed Patient Area, with *p* < 0.001, *p* = 0.014, and *p* = 0.011, respectively.
Carvalho et al., 2021 [47]	662	Cross-sectional; adult Portuguese men and women	COVID-19 confinement levels and sexual functioning	IIEF, FSFI	In men, but not in women, the psychological adjustment for lockdown mediated the association between confinement levels and most domains of sexual functioning. However, even though confinement levels were not directly related to sexual functioning in either gender, psychological adjustment to lockdown was found to predict lower sexual functioning in both men and women.
Hidalgo and Dewitte, 2021 [48]	599	Cross-sectional; adult men and women in the Netherlands	Predictors of sexual function	FSFI, IIEF, SDSS, SDBQ, NSSS, CSI	The results of studies should be interpreted with caution in view of the biases produced by unequal gender distribution, biases of social desirability, volunteer biases, and the particular context of the COVID-19 pandemic. Markers of sexual conservatism were inversely related to lower sexual function and satisfaction, in particular in women, thus possibly representing important targets for treatment. Whereas female sexuality seemed more contextual than male sexuality, improving the general relationship climate may enhance sexual satisfaction in both genders.
Karakas et al., 2020 [49]	135	Cross-sectional; Turkey, adult pregnant women	Sexual function during the COVID-19 pandemic	FSFI	Among non-pregnant participants, 68.9% were diagnosed with sexual dysfunction based on FSFI scores. When comparing pregnant versus non-pregnant subjects, pregnant women had a significantly lower FSFI score in pregnancy (*p* = 0.002). Higher chances of sexual dysfunction were observed among those with a university degree, multipara, and those in the third trimester of pregnancy (*p* = 0.030, *p* = 0.029, and *p* = 0.001, respectively). Also, FSFI scores were significantly higher in women with planned pregnancies compared to those with unplanned pregnancies (*p* = 0.001).
Denizli et al., 2021 [50]	188	Cross-sectional; Turkey, adult women	Depression and sexual function among pregnant and non-pregnant women	ASEX, BDI	Depression scores were similar between pregnant and non-pregnant women, *p* = 0.846, but significantly higher among women of lower economic status, *p* = 0.046, and those who lost income during the pandemic, *p* = 0.027. Sexual dysfunction, measured by the ASEX, was more prevalent among women with lower levels of schooling and income (*p* < 0.05). ASEX scores were also higher in women who experienced greater income loss during the pandemic. Additionally, pregnant women had a significantly higher rate of sexual dysfunction compared to non-pregnant women.
Fang et al., 2021 [51]	612	Cross-sectional; adult Chinese men	Sexual life or sexual function according to changes in lifestyle during the coronavirus pandemic	IIEF-5, PEDT	Around 8.4% and 8.5% of participants reported worsened erectile function or ejaculation control ability, while 31.9% and 17.9% showed decreased IIEF-5 scores or increased PEDT scores. Those with deteriorated erectile function and decreased IIEF-5 scores had higher anxiety (GAD-7) and depression (PHQ-9) scores after the pandemic, along with decreased sexual activity and physical exercise. Participants who had worsened ejaculation control and increased PEDT also had higher anxiety and depression scores. Reduced sexual frequency was associated with reduced income, increased anxiety, and depression. Married participants had improved depression and increased sexual activity.
Sotiropoulou et al., 2021 [52]	299	Cross-sectional; adult Greek men and women	Sexual function and relationship quality of couples during the quarantine	IIEF, FSFI	Most respondents claimed that sexual function was not significantly affected or only minimally affected. Anxiety and mood deficits increased only in those who did not have access to their partner. Couples who were in a steady relationship and lived together, especially childless couples, were more satisfied with their sexual activity and felt more emotionally secure.
Bazyar et al., 2021 [53]	11 studies	Systematic review; Iran, adult men and women	Sexual function and domestic violence	-	The COVID-19 pandemic saw an increased rise in domestic violence, and all sexual function average scores were reported as being poorer than in the period prior to the pandemic.
Salama and Blgozah, 2021 [54]	3	Cross-sectional; Egypt, men who recovered from the COVID-19	Impact of COVID-19 on sexual function	IIEF, BDI, hormonal analyses	Compared with their condition before catching the infection, the men showed, in different degrees, a decline in all aspects of sexual function as assessed by the international index of erectile function. They started to develop premature ejaculation or an existing condition was exacerbated according to the premature ejaculation diagnostic tool scoring. Beck’s depression inventory revealed deterioration of the men’s moods up to severe depression. The sex-related hormones (testosterone—total and free, luteinizing hormone, follicle-stimulating hormone, prolactin, and estradiol) of these men were within normal levels.
Quintana et al., 2024 [55]	2555	Cross-sectional; adult Chilean men and women	Variations in partnered and solo sexual behavior and their relations with sexual function	FSFI, IIEF	Partnered sexual behaviors like intercourse and dating were associated with lower function scores, mainly in satisfaction and desire. Among women, the reduction in the practices of sexual activities such as sexting, foreplay, and masturbation increased the chances of sexual dysfunction in desire, arousal, and satisfaction. Meanwhile, for men, decreased foreplay and intercourse were linked with greater risks of erectile dysfunction and lesser sexual satisfaction. However, men who increased behaviors like foreplay, sexual fantasies, and intercourse saw an improvement in sexual function. The changes in sexual function ranged from small to moderate.
Arafat et al., 2020 [56]	120	Cross-sectional; married Southeast Asian men and women	Changes in sexual behaviors during the lockdown	Survey	Prior to the lockdown, 76.7% of respondents reported having sexual intercourse with their spouse one to five times a week. After the lockdown began, 72.5% continued this frequency. About 45% of respondents indicated that the lockdown impacted their sexual life, while 50% reported positive changes in emotional bonding due to the lockdown.
Hammound et al., 2020 [57]	940	Cross-sectional; adult Australian gay and bisexual men	Impact of physical distancing on the sexual behavior	Survey	Most participants, 88.3%, had sex with men in the six months preceding COVID-19. Of the 587 men, 62.4% who reported sex with casual partners before the pandemic, whereas only 93, 15.8%, reported continued casual sex since the start of the pandemic, which represents an 84.2% reduction.
Lehmiller et al., 2020 [58]	1555	Cross-sectional; USA, adult men and women	Impact on their intimate lives	Survey	While almost half of the participants said their sex life was worse, one in five developed and expanded their sexual repertoire during quarantine by trying new activities like sexting, new sexual positions, and sharing sexual fantasies. Trying new things was related to a younger age, living alone, and higher levels of stress and loneliness. Those adding new activities were three times more likely to report that their sex life was improved.
Rodriguez et al., 2021 [59]	303	Cross-sectional; USA, romantically involved men and women	Benefits of COVID-19 on sexual lives	Survey	The changes in lifestyle were related to negative changes in sexual life, not related to the desire to spend time with a partner, but were positively associated with the quality of the relationship. Among highly COVID-19-infected participants who were afraid, lifestyle changes increased sexual desire, which was connected with positive changes in their sex life and with a desire to spend time with a partner, though not with the overall relationship quality. These findings persisted after controlling for the anxiety linked to the pandemic and demographic variables.
Gleason et al., 2021 [60]	1051	Cross-sectional; adult men and women in USA	COVID-19 pandemic impact on sexual behavior, relationship satisfaction, and partner violence	Survey	The pandemic was associated with a slight decline in partnered sexual activities, while men increased masturbation and pornography use. There was no significant change in relationship satisfaction or intimate partner violence, but slight declines in sexual pleasure were experienced by both genders, while women also reported reduced sexual desire. Casual sex, hookups, and the number of partners declined most, while sexual enjoyment was notably reduced. Changes were predicted by major depressive disorder, relationship status, and views about social distancing. Fewer than half of those who had casual sex before the pandemic stopped completely, with a 6–7-week delay before resuming.
Tan, 2022 [61]	409	Longitudinal design; Singapore, heterosexual married female participants	Sexual health and well-being changes pre- and during the pandemic	Survey	Compared with the pre-pandemic period, the prevalence of participants not having marital sex within a week remained unchanged, but the frequency of sexual activity in a week increased, with a more even distribution of sexual activity between weekdays and weekends. Stress, fatigue, and marital satisfaction predicted both sexual inactivity and frequency.
Wang et al., 2020 [5]	1210	Cross-sectional; adult Chinese men and women	Psychological impact during the initial stage of the COVID-19 outbreak	IES-R, DASS-21	Over half of respondents (53.8%) reported a moderate to severe psychological impact from the outbreak, with 16.5% experiencing moderate to severe depressive symptoms, 28.8% moderate to severe anxiety, and 8.1% moderate to severe stress. Most participants (84.7%) spent 20–24 h at home, and 75.2% were concerned about family members contracting COVID-19. Female gender, student status, physical symptoms (e.g., myalgia, dizziness), and poor self-rated health were associated with higher psychological impact and greater stress, anxiety, and depression. Access to correct health information and precautionary measures were related to a lower psychological impact and lower levels of stress.
Balzarini et al., 2022 [62]	4993	Cross-sectional; Global North countries, adult men and women	Association between multiple stressors and sexual desire	UCLA Loneliness Scale, GMPS, PHQ-4	There was a positive association between financial concern and worry and increased sexual desire during the initial period of the pandemic, whereas stress was related to lower desire. In the subset of participants for whom biweekly monitoring was available, times of greater stress, loneliness, financial strain, or worry were associated with greater depressive symptoms, which in turn were associated with lower sexual desire. The findings suggest that while initially, social isolation and stress had mixed effects on sexual desire, over time, heightened pandemic-related stressors were associated with lower sexual desire, partly due to increased depressive symptoms.
Gillespie et al., 2021 [63]	789	Longitudinal design; UK, adult men and women	Social distancing effects on loneliness, emotion regulation, and self-regulation during lockdown	Survey	There was no overall increase in using sex as a coping mechanism during lockdown compared with beforehand. About 30% of participants reported an increase, 29% reported a decrease, and 41% reported no change. Regression analyses showed that being younger, male, and having greater emotion dysregulation were related to higher use of sex for coping. Moreover, younger age, male gender, not living alone, and less adherence to social distancing were associated with coping using sex in ways involving rape/violence themes during lockdown.
Mollaioli et al., 2021 [64]	6821	Case-control study; adult Italian men and women	Impact of social distancing on the psychological, relational, and sexual health	GAD-7, PHQ-9, DAS, IIEF, FSFI, Orgasmometer	During the lockdown, sexually active participants manifested significantly lower anxiety and depression scores. Regression analysis suggested that gender, sexual activity, and living without a partner during lockdown significantly influenced these scores. Logistic regression analysis revealed that sexual abstinence during the lockdown period heightened anxiety and depression. On the other hand, structural equation modeling revealed that an active sexual life played a protective role in psychological distress, being beneficial both for mental health and for relational and sexual health, with a direct and indirect positive influence, for both males and females.
Cedro et al., 2022 [65]	301	Cross-sectional design; adult Italian men and women	Bodily and sexual experience, before and during COVID-19 lockdown	SAI, Body Uneasiness Test (BUT)	The ANOVA showed that, compared to men, women scored worse on body image avoidance, depersonalization, overall body image quality, sexual arousability, and sexual anxiety. In the between-time comparison, significant correlations between sexual arousal function and changes in body image were present only in women. These correlations, which were weak and scattered before the lockdown, became strong and frequent during the lockdown.
Herbenick et al., 2023 [66]	3743	Cross-sectional design; USA, adult men and women, nationally representative sample	Prevalence, frequency, and reasons to masturbate and masturbate others	Survey	The most common reasons given for masturbating included pleasure, being “horny,” relief of stress, and to relax. The most frequently cited reasons given for not masturbating included a lack of interest, being in a committed relationship, and it going against their moral/religious views. Women who desired more frequent partnered sex were 3.89 times (95% CI: 2.98, 5.08) more likely to report a higher past-year masturbation frequency, while men with the same desires were 4.40 times (95% CI: 3.41, 5.68) more likely to report the same. Those desiring no change in their partnered sex frequency reported a lower masturbation frequency.
Fotinos et al., 2024 [67]	-	Review of empirical studies assessing pornography consumption	Pornography consumption impact on sexual function post-pandemic	-	There was no evidence of sexual dysfunction linked to pornography consumption either during or after the pandemic, despite increased use in lockdowns. In fact, pornography use and solo sex activities served as an alternative to regular sexual behavior during this stressful period and helped reduce psychosocial stress. Those who stayed sexually active reported lower anxiety and depression and better relational health compared to non-sexually active individuals.
Sharma and Subramanyam, 2020 [68]	282	Cross-sectional design and qualitative interview; LGBT adults	Psychological influence of the lockdown	GAD-7, CESD-D, IAT, CPC scale	Anxiety was higher among LGBT adults, individuals in high-risk groups (those with comorbidities), and those with a history of depression or loneliness. LGBT adults also reported using more pornography than heterosexuals during the lockdown. Qualitative data suggested that LGBT individuals may have used pornography and masturbation as coping mechanisms due to limited access to sexual partners and societal stigma surrounding homosexuality. The findings both at a qualitative and quantitative level revealed that frequent communications with family members during the lockdown helped to strengthen social relationships and to enhance social empathy.
Qaderi et al., 2023 [69]	30 studies	Systematic review and meta-analysis in Iran; adult men and women	Changes in sexual function and behaviors during the COVID-19 pandemic	-	Sexual activities were significantly reduced for both women and men in the COVID-19 pandemic, where sexual function declined for both genders but more so in women. A reduction in sexual desire and arousal was also found, though greater in women. A meta-analysis showed a significant decline in sexual satisfaction during the COVID-19 pandemic. Specific changes in sexual behaviors were reported, with an increase in masturbation and use of sex toys being the most prevalent.
Banerjee and Nair, 2020 [70]	26 studies	Narrative review; LGBTQ adults	Effect of COVID-19 pandemic on LGBTQ people’s overall health and well-being	-	Factors at the root of these disparities in health outcomes include immunocompromised states, increased comorbidities, including sexually transmitted diseases, chronic medical disorders, and substance abuse, poor access to healthcare, stigma, social discrimination, and economic constraints. These factors lead to under detection of viral loads, increased risks regarding COVID-19, decreased help-seeking behavior, and inequality in healthcare and legal services. These challenges also contribute to poor emotional and psychosexual well-being, with increased risks for psychiatric disorders and suicidality.
Kara et al. [71]	-	Phenomenological design	Changes in the sexual behavior	-	Feeling such as the need for emotional support, loneliness, devaluation, and helplessness were experienced, and quarantine and curfew restrictions affected sexuality and sexual behaviors. In addition, LGBTQ+ people’s standards changed when finding partners during the pandemic process. The use of sex toys increased, the sensitivity toward self-care and hygiene rules increased, the tendency to have sexual fantasies changed, the sexual behavior of people in their social environment changed, and disruptions in health services posed negative effects.
Odabas al. [72]	518	Cross-sectional design, Turkish married men and women	Changes in the sexual behavior	Survey	18.2% of the married individuals participating in the study had decreased sexual desire during the pandemic, and 49.2% wanted to have a child before the pandemic, but this rate decreased to 19.2% during the pandemic. Sexual behaviors of married individuals changed during the COVID-19 pandemic.
Cascalheiraet al. [73]	565	Cross-sectional design, UK, men and women	Changes in the sexual behavior	Survey	34.3% engaged in more sexual fantasizing during lockdown; women were more likely than men to report this increase. The living context and relationship status were predictors of increased fantasizing. 30.44% reported an increase in at least one solitary sexual practice. This increase was associated with an increase in sexual fantasizing and also with increased pornography consumption. 19% of participants reported an increase in pornography use, with men being more likely than women to report this increase. Participants mostly attributed their increases to boredom, increased free time, and replacing partnered sex.
Storer et al. [74]	831	Cross-sectional design, Australian gay and bisexual men	Trends in sexual behaviors in the COVID-19 context	Survey	Most identified as gay (89.0%) and 10.2% were living with HIV. There was an overall increase in the mean weekly number of non-committed relationship partners (0.53–0.90, *p* < 0.001). The state of Victoria experienced a significantly extended COVID-19 outbreak, accompanied by severe lockdown restrictions. In response, Victorian men’s partner numbers shifted three times, while elsewhere there was an overall gradual increasing trend.
Jiang et al. [75]	806	Cross-sectional design, Australian men and women	Impact of COVID-19 lockdown on drug consumption and sexual behaviors	Survey	Some participants increased drug usage from 2019 to 2020: 25% for alcohol, followed by marijuana (11%), other drugs (8.9%), and tobacco (7%). Risk factors for increasing use included women who have sex with women (WSW) and unsecure employment; protective factors included being in a relationship. The education level was a risk factor for tobacco use, but a protective factor for alcohol use. There was a strong association between drug use and high-risk sexual behaviors. Multivariable analysis found no significant difference in patterns of drug use in Victoria (lockdown) relative to other states (without lockdown).
Hensel et al. [76]	1010	Cross-sectional design, USA men and women	Changes in solo and partnered sexual behaviors	Survey	Adults experienced shifts in sexual behaviors: unpartnered individuals increased sexting, while unemployed individuals reported more oral sex and explicit material use. Partnered affection decreased for those not cohabiting. Household size and children had a minimal impact. Continued research is vital to understand how adults adapted their sexual lives amid ongoing pandemic-related constraints and changes.
Koos et al. [77]	N-T1 = 1747; N-T2 = 656; N-T3 = 411	Cross-sectional design, Hungarian men and women	Potential changes in addictive and problematic behaviors over time	BSMAS, IGDT-10, PGSI, PPCS-6, CSBD-19	Latent growth curve models showed that the sample varied in their initial scores, but there were no significant changes over time in any of the examined behaviors, except for compulsive sexual behavior disorder, which demonstrated a small but significant increase (i.e., positive and significant slope factor). However, the rate of this change was negligible. Overall, there were no noteworthy changes over time regarding any of the examined addictive and problematic behaviors.

A longitudinal study comparing outcomes at three time points (T0: at the start of the pandemic, T1: 12 days after T0, and T2: one month after the end of restrictions in 2020) revealed a significant reduction in the proportion of sexual dysfunction in both men and women between T1 and T2 [6]. Interestingly, when comparing women who were not infected with SARS-CoV-2 to those who either were infected or experienced a long-term SARS-CoV-2 infection (or long COVID), Seehuus et al. [41] showed who women that were not infected scored higher in desire, arousal, lubrication, and satisfaction subscales of the FSFI than both SARS-CoV-2 groups, whereas those who experienced a long-term SARS-CoV-2 infection scored significantly lower in arousal, lubrication, and orgasm, as well as experienced more pain than those who were infected only once.

These findings have been corroborated by literature reviews and meta-analytical studies from various countries [42], which report a greater detriment to female sexual function [15], particularly in the dimensions of desire and satisfaction. Likewise, Ates et al. [40] reported worse sexual function during the pandemic compared to before among older male full-time workers. 

### 3.3. According to What Sexual Function Changed During the Pandemic

Sexual dysfunction during the SARS-CoV-2 pandemic has been significantly associated with various demographic and mental health variables [43]. Pérez et al. [44] demonstrated that individuals with high post-traumatic stress disorder symptoms were more likely to experience sexual dysfunction, with single individuals being 74% more likely to report dysfunction. Among women, sexual dysfunction was worse for those working outside the home, holding higher education degrees, who were multiparous [33], who were older, and who experienced higher levels of stress, anxiety, and depression [45]. In men, sexual dysfunction has been associated with being a medical professional, being married, working with SARS-CoV-2 patients [46], and experiencing greater stress, anxiety, and depression [43]. During the initial lockdown in Portugal, psychological adjustment mediated the relationship between confinement levels (i.e., from one to seven, ranging from “not confined at all” to “strictly confined”) and most domains of male sexual functioning, but not female functioning [47]. Hidalgo and Dewitte [48] demonstrated that, among Ecuadorians in stable relationships, lower female sexual function scores were significantly associated with higher levels of dysfunctional sexual beliefs, lower sexual double standards, and lower sexual and relationship satisfaction. In contrast, lower male sexual function scores were associated solely with reduced sexual satisfaction. Karakas et al. [49] and Denizli et al. [50] found that pregnant women without SARS-CoV-2 exhibited higher levels of sexual dysfunction compared to non-pregnant women, with scores significantly worse.

Other studies report that male and female sexual function indices were not significantly or directly affected for certain individuals in the sample or during specific periods of the pandemic [51,52]. However, such reports appear to be less common. In fact, a systematic review identified consistent reductions in sexual function scores for both men and women attributed to the pandemic and social isolation measures [53].

### 3.4. Changes in Sexual Behavior Patterns

During the pandemic, sexual behavior patterns varied, demonstrating a range of impacts [55]. A survey in three Middle Eastern countries revealed that 55% of respondents felt their sexual life was unaffected by the lockdown, while others experienced a decrease or even an increase in sexual activity frequency [56]. Hammoud et al. [57] found a dramatic reduction in sexual behaviors among gay and bisexual men in Australia, with 62.4% reporting decreased casual sexual encounters due to concerns about SARS-CoV-2 transmission. Lehmiller et al. [58] reported that over 50% of a sample of 1559 Americans experienced a decline in their sexual life compared to pre-pandemic levels, while 20% introduced new sexual activities to their repertoire. Among those in a relationship during the pandemic, Rodríguez and Lehmiller [59] showed that lifestyle changes were associated with negative impacts on sexual life but positively correlated with sexual desire only in those with high levels of fear of COVID-19 infection. Gleason et al. [60], through a national sample from the U.S. (*N* = 1051), reported that among various sexual behaviors, casual sex experienced the most significant decline. Less than 50% of participants who engaged in casual sex before the pandemic continued this behavior after its onset. However, as in many other studies, for each sexual behavior, there was a substantial proportion of participants—sometimes even greater—whose frequency of behavior did not change or even increased during the pandemic (ranging from 5% to 38% of the sample) [60]. For instance, Tan [61] found that, compared to pre-pandemic levels, the proportion of married women with no sexual activity within a week remained stable, while weekly sexual frequency increased, showing patterns influenced by levels of stress, fatigue, and marital satisfaction.

### 3.5. Why Sexual Behaviors Changed During the Pandemic

Sexual behaviors during the COVID-19 pandemic exhibited varied changes influenced by a range of sociodemographic, psychological, and contextual factors. Sociodemographic characteristics like gender, sexual orientation, age, relationship status, and employment modulated some behavioral changes. Partnered individuals, especially those cohabitating, generally experienced declines in sexual activity, while non-partnered individuals engaged more in sexting or other virtual sexual practices [39,60], whereas married individuals experienced reduced sexual desire and childbearing motivations during the pandemic [72]. Meanwhile, LGBTQ+ individuals reported significant behavioral shifts, including increased sexual fantasies, partner selection changes, and greater reliance on sex toys, motivated by feelings of loneliness and the constraints of lockdowns [71].

It is well-documented that the pandemic has severely impacted mental health, reflected in heightened symptoms of conditions such as stress, anxiety, and depression [5]. However, Balzarini et al. [62] found that greater economic concerns were associated with greater sexual desire, whereas general stress was linked to lesser sexual desire. Similarly, Gillespie et al. [63] reported that 30% of survey participants in the UK used sex as a coping mechanism during lockdowns, 29% did not, and 41% reported no changes in their sexual behaviors. Furthermore, anxiety and depression symptoms were found to be lower in individuals who remained sexually active during the pandemic and higher in those who were not [64]. The interaction of depressive symptoms and impulsivity were linked to breaking shelter-in-place orders for partnered sexual activities [38]. Additionally, other factors associated with sexual function, such as body image, were significantly affected, directly impacting sexual anxiety and arousability. These effects, sporadic before the pandemic, became frequent and more intense during the pandemic [65]. Some individuals turned to masturbation and sexual activity for temporary relief from negative emotions during the pandemic, as well as pleasure and “feeling horny” [66], where boredom and increased free time drove a rise in pornography use and masturbation [73]. LGBTQ individuals reported higher pornography use than heterosexuals during lockdown [67,68]. Lehmiller et al. [58] demonstrated that non-binary individuals did not reduce their masturbation rates. Indeed, Qaderi et al. [69] showed through a systematic review that masturbation was one of the most significant and common changes in sexual behavior during the pandemic. Lastly, particular attention is needed on the challenges and difficulties sexual minorities faced during the pandemic—especially at its onset—such as stigmatization, poor access to sexual health services, increased suicide rates, and a heightened risk of psychiatric disorders [70].

Contextual factors, such as the stringency of lockdowns, geographic location, and pandemic-related restrictions, also played a role in shaping sexual behaviors. Severe lockdowns were associated with reduced casual sexual encounters and fewer partners among gay and bisexual men, particularly in regions with extended restrictions and high COVID-19 notification rates [74]. Conversely, in regions with less severe restrictions, changes in sexual behavior were less pronounced [75]. Household composition and employment status were significant, as those living with children or employed in essential roles reported fewer partnered sexual activities, whereas unemployed individuals saw increases in solo activities and consumption of sexually explicit materials [76]. Interestingly, addictive and problematic sexual behaviors, such as compulsive sexual behavior disorder, showed minimal increases, suggesting stability among those without pre-existing issues [77].

### 3.6. Why Sexual Function and Behaviors Changed (or Did Not) During the Pandemic

Gilbert and Barkun [78] describe how, in the late 18th-century Western society, natural disasters were linked to ’deviant’ sexual behaviors. Disasters were often viewed as divine punishment, prompting every social, religious, and legal institution to suppress anything perceived as immoral. This historical context highlights a long-standing association between hypersexuality and catastrophic events.

Two hypotheses attempt to explain why some individuals experienced increases or decreases in sexual desire and behaviors: terror management theory [79] and the dual control model of sexual response [80]. The first hypothesis, based on Ernest Becker’s work on death [81], explores how humans strive to enhance self-esteem as a defense against death-related anxiety. According to terror management theory, individuals facing threatening events such as natural disasters or the SARS-CoV-2 pandemic may experience significant stress and anxiety, triggering a strong desire for intimacy and closeness or fear of abandonment, which can either increase or inhibit sexual desire [59,82]. For example, Birnbaum et al. [83] found that reminders of mortality heightened the desire for casual, non-romantic sex among men. When the encounter was manipulated to have romantic connotations, both men and women exhibited increased sexual desire.

The dual control model of sexual response, on the other hand, adopts a psychophysiological perspective that distinguishes individuals based on their tendency toward excitation or inhibition in response to stimuli. While stressful events may hinder sexual desire and arousal for some, they may encourage or amplify sexual behaviors for others [80]. Men generally exhibit stronger sexual desire than women across various cultures [84], partially explaining why men may experience greater increases in desire and sexual activity in response to the pandemic [85]. Additionally, social distancing measures gave people more free time, fostering boredom, which is believed to lead to hypersexual behaviors [86,87] to escape negative feelings such as loneliness [88], sadness, or boredom [89].

## 4. Discussion

Sexual health is a critical component of human well-being, and ample evidence demonstrates how this pandemic impacted individual, relational, and public sexual health. However, it has been one of the most neglected aspects of health, receiving limited attention and resources. Therefore, this manuscript explored pandemic-related changes in sexual function and behaviors, while also highlighting public health’s role in the context of a pandemic.

The effects of the pandemic on sexual behaviors were diverse [15,34,55,90]. As shown by the thematic analysis, the SARS-CoV-2 pandemic significantly affected sexual health with four themes, with declines in desire, satisfaction, and arousal, especially among women. Partnered sexual activities, like casual sex, decreased, while solo behaviors, including masturbation and pornography use, increased due to restricted partner access and boredom. Mental health challenges, such as stress and anxiety, exacerbated sexual dysfunction but also drove some to use sexual activity as a coping mechanism. Demographic and contextual factors, including gender, relationship status, and socioeconomic conditions, shaped these experiences, with LGBTQ+ individuals facing unique challenges and greater reliance on solitary or virtual sexual practices. These findings reveal the pandemic’s multifaceted impact on sexuality.

In general, decreases in explored sexual behaviors across studies conducted during the pandemic tended to outweigh increases at a ratio of 2:1. It is of particular interest to understand the magnitude of increases, decreases, or stability in dynamics within couples, as well as the effect on couples when one partner experiences an increase in sexual function and behavior while the other experienced a decrease. Furthermore, despite this great deal of variability, most studies reported a decline in sexual desire, frequency, and satisfaction; some people showed an increase, especially in solo sexual activities, where changes in sexual function appeared greater in women compared to men [15]. However, the effect on desire and lubrication appeared minimal [36]. The psychological toll of the pandemic was manifested in increased stress, anxiety, and depression, which often mediated these changes and exacerbated pre-existing sexual health disparities. Such results signal the need to integrate sexual health into public health responses in the context of crises. However, certain populations remain underrepresented in the reviewed literature, particularly LGBTQIA+ individuals. This community faced unique challenges during the pandemic, including limited access to affirming sexual health services, compounded by social stigma and discrimination [16,69]. Addressing these disparities in future research is critical to ensuring equitable sexual health resources. Policies should prioritize inclusive interventions, such as tele-medical services and culturally sensitive sexuality education, to support sexual minorities during public health emergencies.

Further studies are needed to explore sexual function in individuals infected with SARS-CoV-2, both with and without vaccination, as well as post-infection and longitudinal effects. Health and isolation conditions varied widely among individuals, depending on factors such as family situation, employment, socioeconomic status, and stress, anxiety, or depression symptoms [91]. It is plausible that these same factors contributed to changes in sexual function and behaviors during the pandemic [92]. For example, Carvalho et al. [47] demonstrated that confinement not only affected sexual function but also that this impact varied by gender and whether individuals were in a romantic relationship [93]. Furthermore, future research should aim to uncover the personal and contextual variables that shield individuals from significant sexual function impacts during events like the pandemic and associated social distancing measures, as well as elucidate how infection specifically influences sexual function. On this latter point, compared to their pre-infection condition, men reported declines across all dimensions of sexual function, and developed premature ejaculation and depressive symptoms, though their hormone levels remained unaffected [54], whereas women showed a decline in sexual function scores compared to the scores 60 days after discharge [37].

Virtually every dimension of sexual health has the potential to promote physical and mental health, prevent infections, and maintain the “normality” of sex in reproductive and recreational terms during a pandemic like SARS-CoV-2. Therefore, it is the unavoidable responsibility of governments to include policies that enhance sexual health prevention and promotion in public health emergency planning for future pandemics [94,95]. Some strategies and measures to integrate sexual health into public health programs date back more than 20 years [11]. The existing body of knowledge, coupled with recent experiences highlighting the consequences of insufficient sexual health policies, underscores the critical importance of preparedness for future pandemics. A lack of readiness would be a significant oversight.

### 4.1. The Role of Public Health in Sexual Health

A public health policy that fails to address sexual health comprehensively is fundamentally incomplete. This became abundantly clear during the pandemic, especially in its early stages, as evidenced by the lack of information, planning, and implementation of policies that could provide a timely and effective response [17,96,97]. This impact was most apparent in services related to preventing sexually transmitted infections, pregnancy and family planning, abortion access, and providing resources, information, and sexual education [9,78]. When nearly 1200 individuals were surveyed regarding sexual health services in British Columbia, Canada [78], more than half reported needing some form of sexual health service, of which access was either unavailable or delayed. Common barriers included discouragement from seeking non-urgent medical care, fear of contracting COVID-19, and the closure of usual access points. However, alternative models for sexual health services, such as home-based self-collection kits and rapid tests, gained significant traction.

Early on, Hall et al. [98] predicted the risks the pandemic would pose for exercising women’s sexual and reproductive rights, particularly among sexual and gender minorities. They also demonstrated how providing basic sexual health services, such as contraception, could help prevent or mitigate mortality rates. Similarly, the Economic Commission for Latin America and the Caribbean (ECLAC) forecasted the same risks for Latin America, with the potential for significant setbacks [99].

During pandemics, contagion is a central and urgent concern. Consequently, the transmissibility of the virus through sexual secretions and fluids becomes an important issue for sexual and public health. While SARS-CoV-2 is primarily transmitted through respiratory secretions [9], nearly all forms of sexual contact pose a risk of infection. Some studies report no presence of the virus in genital secretions [100,101], while others have detected it in semen [102], vaginal fluids [103], and feces [104] at relatively low proportions. Various factors, such as the timing of sample collection, severity of symptoms, and immunization status, may explain the variability of results. However, in general, the virus appears to have low sexual transmissibility [105].

Other psychological, social, political, and situational factors likely exacerbated the spread of the virus. These include the dissemination of misinformation [106], the suggestion of sexual abstinence through news outlets and social media [107], the lack of a comprehensive and up-to-date body of knowledge on the sexual transmission of SARS-CoV-2 [96], and the overshadowing of sexual transmission discussions by other virus-related issues among authorities and experts. Nevertheless, several scientifically based reports from different perspectives were made available to guide both authorities and the public on what is considered safe and reasonable under different circumstances [8,96,108]. Unfortunately, these resources often arrived months late.

Although the worst of the pandemic has passed, it is essential to learn, document, and remember what actions were taken—and which were not—to prepare for future pandemics. Therefore, what should public health programs suggest in the event of a future coronavirus outbreak? Below, I offer a few considerations for public health professionals.

The SARS-CoV-2 pandemic has underscored the need for planned public health responses capable of addressing systemic inequities, improving communication, and acknowledging the diversity of lived experiences. For instance, marginalized groups, including those with a lower socioeconomic status, limited education, and non-normative sexual diversities, faced disproportionate health disparities, exacerbated by communication inequalities and narrowly framed public health messaging [109,110]. In order to mitigate these inequities, future public health strategies must adopt inclusive, tailored communication approaches that account for linguistic and cultural diversity, leveraging community partnerships to enhance accessibility and trust. Tailored-made efforts addressing multi-cultural populations and those with high numbers of immigrants and refugees have proven to be feasible and successful [111,112]. Moreover, integrating community-driven risk reduction strategies, as demonstrated in the adaptations of gay and bisexual men during COVID-19, can promote adherence while avoiding stigmatization [113]. Policymakers are also encouraged to expand their frameworks to include diverse kinship configurations and address structural barriers such as economic insecurity and healthcare access that disproportionately impact vulnerable populations. Similarly, the response will depend on the conditions and the individuals involved in sexual relations. From the outset, it is the responsibility of health authorities to provide education and alternatives, reducing risks and promoting informed decision-making before or after infection has occurred [9]. This approach can help prevent the spread of the virus, particularly if it is sexually transmissible. Additionally, SARS-CoV-2 has raised significant concerns for pregnant women and families planning to have children. The available literature shows that pregnancy does not alter the severity of SARS-CoV-2 infection in mothers [114], and vertical transmission appears to be infrequent [115]. However, pregnancies in women infected with SARS-CoV-2 are more likely to result in premature births [114]. The primary objectives in such cases are to ensure a safe pregnancy for both the mother and the fetus and to plan or prevent unintended pregnancies, particularly in situations where social distancing is ineffective or unfeasible. Consequently, the American Society for Reproductive Medicine has recommended encouraging vaccination for all women—pregnant or planning pregnancy—once vaccines are deemed entirely safe for pregnant women [116]. Lastly, the pandemic has clearly reduced access to and availability of sexual health services. Quarantines led to disruptions and delays in services such as prescription issuance, contraceptive purchases, family planning, access to abortion clinics, STI testing, treatment, follow-ups, and access to information [94,95]. Evidently, conditions have improved with the availability of vaccines and the incorporation of telemedicine [117]. Only contingency plans and preventive measures, tailored to the modes of virus transmission, can avoid such disruptions, ensuring the health of both patients and healthcare professionals. These efforts must be implemented with a focus on protecting those who are most exposed and vulnerable.

### 4.2. Limitations and Future Directions

It is important to consider that there is a vast degree of variability across studies in terms of study design, populations, psychometric tools used, clinical samples, controlling variables, procedures (e.g., times at which the study was conducted during the pandemic), etc. Whereas this can be construed as a strength of the present study in its analysis of the main trends across a variety of settings, cultures, methodologies, etc., it is also a caveat. For instance, whereas on the one hand, the selected studies stemmed from several different countries, improving the generalizability of the general trend findings of this study, very few of the studies mentioned, analyzed, or discussed the degree to which their questionnaires were psychometrically invariant when comparing groups [118]. To this point, the FSFI has only been shown to have strong measurement invariance when comparing based on sexual orientation [119], age group, and relationship status [21], whereas the IIEF has also proven to be invariant between age groups and relationship statuses [23]. However, the reader should bear in mind that most studies were conducted in a trying time, the pandemic, in which limited resources forced researchers to sacrifice quality in different ways.

The present study is not without limitations. First, the registration of this study protocol was not made available, creating a lack of information, through most of the protocol is part of the manuscript’s Methods section. Furthermore, the results, and consequently the discussion, are limited by the databases consulted and the restriction to English-language studies. Similarly, the reviewed studies were limited by the key terms; while comprehensive, they were not specific. Moreover, the present study did not include qualitative studies and excluded all gray literature, thus limiting the coverage of individual perspectives and experiences.

Future research should address the gaps identified in this review by focusing on the longitudinal effects of the SARS-CoV-2 pandemic on sexual health, particularly among diverse populations and underrepresented groups. Investigating sexual function in individuals infected with the virus, including vaccinated and unvaccinated populations, will provide valuable insights into the interplay between infection, immunity, and sexual health. Additionally, understanding the differential impact of the pandemic across demographic variables, such as gender, socioeconomic status, and relationship type, can guide tailored public health interventions. Studies exploring the resilience factors that protected some individuals from significant sexual health disruptions are also critical, as they may inform strategies to mitigate similar impacts in future crises. Lastly, effort made to integrate sexual health considerations into pandemic preparedness plans, including the use of telemedicine and other service delivery models, is essential to ensure equitable access to sexual health resources during public health emergencies.

## 5. Conclusions

The SARS-CoV-2 pandemic had profound effects on sexual function and behavior, highlighting the varied ways in which individuals respond to stress and a crisis. While some experienced reduced sexual desire and activity, others saw increases, underscoring the complexity of human responses to extreme situations. Integrating sexual health into public health emergency plans is crucial to ensure comprehensive support for well-being during such crises. Future research should focus on understanding the factors that contribute to resilience in sexual health, as well as exploring the long-term consequences of the pandemic on sexual function and behavior. Addressing these aspects will strengthen responses to future public health emergencies.

## Data Availability

Not applicable.

## Supplementary Materials

The following supporting information can be downloaded at https://www.mdpi.com/article/10.3390/healthcare13050559/s1. Supplementary File S1. PRISMA-ScR checklist [120].
